# Influence of Labor Market Disparities on Sex and Gender Inequalities in Cognitive Decline

**DOI:** 10.1093/geroni/igab046.1925

**Published:** 2021-12-17

**Authors:** Justina Avila-Rieger, Precious Esie, Jennifer Manly

**Affiliations:** Taub Institute for Research on Alzheimer's Disease and the Aging Brain, New York, New York, United States, 2 Columbia University, New York, New York, United States

## Abstract

State-level labor market disparities have been linked to health outcomes. The current study examines how labor market disparities may shape different patterns of sex/gender inequalities in cognition across race/ethnicity, place, and time. We leverage cognitive outcome data from multiple cohort and nationally representative longitudinal studies, as well as historical data on labor force participation and occupational status from IPUMS CPS. Multilevel modeling analyses was used to examine heterogeneity in sex/gender inequalities in cognitive trajectories within and between race/ethnicity and U.S. state of birth and determine whether such variability is explained by a state-level labor market opportunity composite. We expect women to demonstrate an advantage over men on cognitive measures. Women’s advantage will be more pronounced in states with a small sex/gender gap in labor market opportunities and less pronounced in states with a large gap. The magnitude of this advantage will be greater for White women compared with Black women.

